# Multiple Myeloma and Glyphosate Use: A Re-Analysis of US Agricultural Health Study (AHS) Data

**DOI:** 10.3390/ijerph120201548

**Published:** 2015-01-28

**Authors:** Tom Sorahan

**Affiliations:** Institute of Occupational and Environmental Medicine, University of Birmingham, Edgbaston, Birmingham B15 2TT, UK; E-Mail: T.M.Sorahan@bham.ac.uk; Tel.: +44-121-414-3644; Fax: +44-121-414-6217

**Keywords:** glyphosate, multiple myeloma, pesticides, prospective cohort study

## Abstract

A previous publication of 57,311 pesticide applicators enrolled in the US Agricultural Health Study (AHS) produced disparate findings in relation to multiple myeloma risks in the period 1993–2001 and ever-use of glyphosate (32 cases of multiple myeloma in the full dataset of 54,315 applicators without adjustment for other variables: rate ratio (RR) 1.1, 95% confidence interval (CI) 0.5 to 2.4; 22 cases of multiple myeloma in restricted dataset of 40,719 applicators with adjustment for other variables: RR 2.6, 95% CI 0.7 to 9.4). It seemed important to determine which result should be preferred. RRs for exposed and non-exposed subjects were calculated using Poisson regression; subjects with missing data were not excluded from the main analyses. Using the full dataset adjusted for age and gender the analysis produced a RR of 1.12 (95% CI 0.50 to 2.49) for ever-use of glyphosate. Additional adjustment for lifestyle factors and use of ten other pesticides had little effect (RR 1.24, 95% CI 0.52 to 2.94). There were no statistically significant trends for multiple myeloma risks in relation to reported cumulative days (or intensity weighted days) of glyphosate use. The doubling of risk reported previously arose from the use of an unrepresentative restricted dataset and analyses of the full dataset provides no convincing evidence in the AHS for a link between multiple myeloma risk and glyphosate use.

## 1. Introduction

Glyphosate [*N*-(phosphonomethyl)glycine] is a broad-spectrum herbicide active ingredient, commonly sold as commercial formulations under a range of Roundup^®^ branded products [1]. Data on the use of glyphosate and many other herbicides, insecticides, and fungicides were collected from 1993–1997 by researchers in a prospective cohort study of some 57,000 licensed pesticide applicators from the US States of Iowa and North Carolina [2]. At enrolment information was also sought on basic demographic data as well as information on smoking history, use of alcohol, and other lifestyle factors. Mortality and cancer incidence data are being collected by the AHS researchers and many publications have already appeared based on these data. The purpose of this secondary analysis is to understand the conflicting findings of De Roos and colleagues that have appeared on the risks of multiple myeloma in those applicators that have used glyphosate [3].

The above paper [3] contains two disparate findings for the risks of multiple myeloma in ever users of glyphosate, with cancer incidence data to the end of 2001: an unexceptional risk in 54,315 applicators in analyses that only adjusted for age (rate ratio or relative risk (RR) 1.1, 95% confidence interval (CI) 0.5 to 2.4) and a non-significantly elevated risk in 49,211 applicators in analyses that also adjusted for level of education, smoking history, use of alcohol, history of cancer in first degree relatives, State of residence, and use of ten other pesticides (RR 2.6, 95% CI 0.7 to 9.4). The second finding, based on the smaller dataset, excluded applicators with missing data for any of the variables included in the analysis. Both findings excluded applicators diagnosed with cancer prior to enrollment into the AHS and applicators who did not know whether or not they had ever used glyphosate. It is most unusual to observe such differences in risk estimates derived from the same study (RRs of 1.1 and 2.6), and whilst the higher risk is not significantly elevated, glyphosate users, producers of glyphosate, and regulatory agencies need to understand how these differences came about and which result should be given more importance. These disparate findings have been highlighted previously in published correspondence [4,5].

Little is known about the causes of multiple myeloma. A recent review published in six parts by the International Agency for Research on Cancer (IARC) included an evaluation of the target sites for all the IARC Group I carcinogens [6,7,8,9,10,11]. The only carcinogen that showed sufficient evidence for producing multiple myeloma in humans was gamma radiation [9]. Limited evidence was found for exposures to benzene and ethylene oxide [11]. There have, however, been a number of studies reporting excess risks of multiple myeloma in connection with specific industries including farm workers [12,13].

## 2. Materials and Methods

The secondary data file analysed in this report was kindly provided by AHS researchers and included no information on identifying particulars (names, addresses, dates of birth, or social security numbers). The file was not a copy of the entire AHS dataset but rather a copy of those variables used by De Roos* et al.* [3], except that data on race, State of residence, and applicator type were not supplied because of concerns that these variables could lead to the identification of study participants, and that any such identification would constitute an unwarranted invasion of privacy. AHS researchers supplied an informative description of the file and the file was found to be internally consistent as well as consistent with data descriptions supplied earlier [3]. All subjects gave their informed consent for inclusion before they participated in the AHS study and ethics approval for the original data collection by AHS researchers was obtained from the Institutional Review Board of the National Institutes of Health; this secondary analysis was conducted in accordance with the Declaration of Helsinki and the protocol was approved by the University of Birmingham Science, Technology, Engineering and Mathematics Ethical Review Committee (project code ERN_11-0758, 20 July 2011).

Cohort enrolment, and follow-up for the period 1993–2001, have been described previously [2,3]. In summary, data on lifestyle factors and use of pesticides were collected from 57,311 private and commercial pesticide applicators from Iowa and North Carolina. Previous analyses [3] were carried out on three subsets of data. The first set comprised 54,315 applicators and this was obtained by excluding applicators with any cancers diagnosed before enrolment (*n* = 1074), applicators lost to follow-up (*n* = 298), who had missing data for age at enrolment (*n* = 7), or who provided no information on whether they had ever used glyphosate (*n* = 1678); these exclusion categories are not mutually exclusive. The second set of data comprised 49,211 applicators and this was obtained by a further exclusion of applicators with missing data on level of education (a surrogate for socio-economic status) (*n* = 1296), smoking history (*n* = 1783), or use of alcohol (*n* = 2616); information was complete for State of residence (not available to current analysis), and family history of cancer in first degree relatives. The third set of data comprised 40,719 applicators and this was obtained by a further exclusion of applicators with missing data on either use or estimated cumulative exposure days for 2,4-D (2,4-dichlorophenoxy acetic acid) (*n* = 1202), alachlor (*n* = 3142), atrazine (*n* = 917), metolachlor (*n* = 2925), or trifluralin (*n* = 3203), missing data on ever-use of benomyl (*n* = 2467), maneb (*n* = 2674), paraquat (*n* = 2737), carbaryl (*n* = 1828), or diazinon (*n* = 2701). 

This analysis aimed to examine findings in as full a dataset as possible and some analyses have also been carried out on a larger fourth set of 55,934 applicators, a category that does not exclude applicators with missing data on ever-use of glyphosate but only applicators with cancers diagnosed before enrolment, applicators lost to follow-up, or who had missing data for age at enrolment.

Poisson regression was used to estimate RRs and 95% CIs associated with glyphosate exposure metrics, with and without adjustment for other variables [14]. Each variable under analysis was classified into levels or categories. The analytical approach for the full dataset was to have a “not known/missing” category for each variable so that analyses of all available cases could be maintained. However it was necessary to ensure that there was at least one case of multiple myeloma in each level of each variable for the regression to successfully calculate RRs. There were no cases of multiple myeloma in those applicators with “unknown use of 2,4-D”; such applicators were combined with those reporting “no use” to create a new category of “no claim of use”. There were no cases of multiple myeloma in those applicators with “unknown level of education”; such applicators were combined with those reporting no education beyond high school. All significance tests were two-tailed and tests for trend (where applicable) were calculated by scoring the levels of a variable and treating the variable as unfactored. All analyses were performed with the EPICURE statistical software [15], using the double precision DOS version 2.12 of DATAB and AMFIT, dated March 2002.

## 3. Results

Table 1 shows RRs for multiple myeloma in terms of reported ever-use of glyphosate. Findings are shown for the first three sets of applicators, there were 32 cases of multiple myeloma in Set 1, 26 cases in Set 2, and 22 in Set 3. There was no adjustment for gender in the findings in this Table, in order to follow the approach adopted by De Roos and colleagues [3]. The first column of RRs includes statistical adjustment for age at enrolment only (see Table 1 footnotes for levels), the second column includes additional adjustments. The Table summarises the results of five separate analyses. None of the point estimates is statistically significant and in Set 1, the largest data set, the RR for ever-use of glyphosate is close to unity (RR 1.08, 95% CI 0.48 to 2.41). The point estimates of risk for the smaller datasets show an approximate doubling of risk irrespective of whether adjustment for other variables is carried out; the largest RR is shown for the fully adjusted model of the smallest dataset.

**Table 1:** Risks of multiple myeloma from the AHS in relation to use of glyphosate in three sets of data.

Set ^a^	Subjects		Multiple Myeloma Cases (*n*)	RR ^b^	(95% CI)	RR	(95% CI)
1	Non-users	13,280	8	1.0			
	Users	41,035	24	1.08	(0.48 to 2.41)	--	--
	Total	54,315	32				
2	Non-users	11,881	4	1.0			
	Users	37,330	22	1.91	(0.66 to 5.53)	2.07 **^c^**	(0.71 to 6.04)
	Total	49,211	26				
3	Non-users	9809	3	1.0			
	Users	30,910	19	2.21	(0.65 to 7.48)	2.79 **^d^**	(0.78 to 9.96)
	Total	40,719	22				

**^a^** Set 1 comprised 54,315 applicators excluding those with any cancers diagnosed before enrolment, applicators lost to follow-up, who had missing data for age at enrolment, or who provided no information on whether they had ever used glyphosate. Set 2 comprised 49,211 applicators and further excludes applicators with missing data on level of education, smoking history, or use of alcohol. Set 3 comprised 40,719 applicators and further excludes applicators with missing data on either use or estimated cumulative exposure days for 2,4-D (2,4-dichlorophenoxy acetic acid), alachlor, atrazine, metolachlor, or trifluralin, and missing data on ever-use of benomyl, maneb, paraquat, carbaryl, or diazinon. **^b^** Adjusting for age at enrolment only (<50 years, 50–59 years, 60–69 years, ≥70 years); **^c^** Further adjustment for cigarette smoking (never smoker, ≤12 pack years, >12 pack years) use of alcohol in year before enrolment (none, <72 drinks, ≥72 drinks, history of cancer in first degree relatives (no/yes), and level of education (≤ high school and not known, > high school); **^d^** Further adjustment for level of use of 2,4-D, alachlor, atrazine, metolachlor, or trifluralin (all classified in terms of none, ≤ median reported cumulative days of use, >median reported cumulative days of use), and ever use of maneb, paraquat, carbaryl, diazinon and benomyl (all classified in terms of yes/no).

Table 2 also shows RRs for multiple myeloma in terms of reported ever-use of glyphosate for the 54,315 applicators in Set 1, with a total of 32 cases of multiple myeloma. The first column of RRs summarises the findings from 15 separate analyses in which each variable is analysed simultaneously with age at enrolment and gender. The second column of RRs summarises the findings from a single analysis in which all 15 variables are analysed simultaneously, also adjusting for age at enrolment and gender. The RR for ever-use of glyphosate, with adjustment for age at enrolment and gender only, is close to unity (RR 1.12, 95% CI 0.50 to 2.49). The RR for ever-use of glyphosate is little changed with additional adjustment for all 14 other variables (RR 1.24, 95% CI 0.52 to 2.94). None of the point estimates of risk in the Table are statistically significant and there is no suggestion that smoking or alcohol use are risk factors for multiple myeloma.

**Table 2:** Estimated risks of multiple myeloma from the AHS study in relation to use of pesticides and other variables (32 cases (29 males, 3 females) in 54,315 applicators, Set 1 data **^a^**).

Variable	Cases	Separate Analysis ^b^		Simultaneous Analysis ^c^	
		RR	(95% CI)	RR	(95% CI)
*Glyphosate use*					
Never	8	1.0		1.0	
Ever	24	1.12	(0.50 to 2.49)	1.24	(0.52 to 2.94)
*Smoking*					
Never	17	1.0		1.0	
≤12 pack years	5	0.63	(0.23 to 1.72)	0.66	(0.24 to 1.83)
>12 pack years	7	0.66	(0.27 to 1.60)	0.66	(0.27 to 1.65)
not known	3	1.77	(0.52 to 6.10)	1.57	(0.43 to 5.69)
*Alcohol in year before enrollment*					
None	19	1.0		1.0	
<72 drinks	6	0.47	(0.18 to 1.18)	0.52	(0.20 to 1.36)
≥72 drinks	4	0.47	(0.16 to 1.41)	0.55	(0.18 to 1.71)
not known	3	0.94	(0.28 to 3.18)	1.73	(0.42 to 7.15)
*Family history of cancer*					
No	15	1.0		1.0	
Yes	17	1.47	(0.74 to 2.95)	1.55	(0.76 to 3.16)
*Education*					
≤high school (or not known)	26	1.0		1.0	
>high school	6	0.48	(0.19 to 1.20)	0.50	(0.20 to 1.26)
*2,4-D*					
no claim **^ d^**	12	1.0		1.0	
≤median use	10	0.57	(0.24 to 1.34)	0.46	(0.18 to 1.15)
>median use	10	0.72	(0.30 to 1.72)	0.57	(0.21 to 1.60)
*Alachlor*					
None	13	1.0		1.0	
≤median use	7	1.06	(0.42 to 2.70)	0.91	(0.33 to 2.55)
>median use	8	1.26	(0.51 to 3.09)	1.10	(0.38 to 3.14)
not known	4	0.89	(0.29 to 2.76)	0.71	(0.13 to 4.04)
*Atrazine*					
None	8	1.0		1.0	
≤median use	11	1.61	(0.63 to 4.12)	1.68	(0.61 to 4.67)
>median use	12	1.77	(0.69 to 4.51)	2.02	(0.67 to 6.10)
not known	1	1.44	(0.18 to 11.6)	1.16	(0.14 to 9.86)
*Metolachlor*					
None	14	1.0		1.0	
≤median use	7	1.56	(0.62 to 3.91)	1.73	(0.62 to 4.77)
>median use	6	1.38	(0.53 to 3.65)	1.57	(0.51 to 4.82)
not known	5	1.28	(0.46 to 3.58)	2.84	(0.59 to 13.8)
*Trifluralin*					
None	16	1.0		1.0	
≤median use	9	1.16	(0.51 to 2.65)	1.07	(0.43 to 2.63)
>median use	4	0.59	(0.19 to 1.77)	0.47	(0.14 to 1.58)
not known	3	0.59	(0.17 to 2.04)	0.52	(0.11 to 2.50)
*Maneb use*					
Never	24	1.0		1.0	
Ever	4	1.12	(0.39 to 3.23)	1.56	(0.46 to 5.30)
not known	4	0.81	(0.28 to 2.37)	2.46	(0.33 to 18.3)
*Paraquat use*					
Never	21	1.0		1.0	
Ever	7	0.96	(0.41 to 2.27)	0.83	(0.33 to 2.10)
not known	4	0.74	(0.25 to 2.19)	0.99	(0.15 to 6.49)
*Carbaryl use*					
Never	7	1.0		1.0	
Ever	23	1.85	(0.79 to 4.34)	2.48	(0.99 to 6.21)
not known	2	0.85	(0.17 to 4.15)	1.07	(0.13 to 8.91)
*Diazinon use*					
Never	21	1.0		1.0	
Ever	7	0.60	(0.26 to 1.41)	0.50	(0.20 to 1.25)
not known	4	0.66	(0.22 to 1.96)	0.78	(0.11 to 5.55)
*Benomyl use*					
Never	26	1.0		1.0	
Ever	3	0.72	(0.22 to 2.39)	0.51	(0.13 to 2.03)
not known	3	0.58	(0.17 to 1.95)	0.23	(0.02 to 2.46)

**^a^** Set 1 comprised 54,315 applicators excluding those with any cancers diagnosed before enrolment, applicators lost to follow-up, who had missing data for age at enrolment, or who provided no information on whether they had ever used glyphosate. **^b^** Column summarises 15 separate analyses in which each variable is adjusted for age at enrolment (<50 years, 50–59 years, 60–69 years, ≥70 years) and gender; **^c^** Single simultaneous analysis of all 15 variables, also adjusting for age at enrolment and gender. **^d^** None and not known combined.

Table 3 shows RR estimates for multiple myeloma in terms of levels of reported cumulative days of glyphosate use and levels of estimated intensity-weighted exposure days for the 54,315 applicators in Set 1. The latter exposure metric is in arbitrary units and has been developed by AHS researchers to make use of data collected on work practices. For each exposure metric three sets of RRs have been calculated; firstly adjusting for age at enrolment and gender, secondly with additional adjustment for cigarette smoking, use of alcohol, family history of cancer, and level of education, and thirdly with additional adjustment for use of ten other pesticides. The Table summarises findings from six separate analyses. Two tests for trend were applied to each of these analyses, the first scored the levels of cumulative exposure as 1–4, the second scored the level by mean values (shown in Table 3 footnotes). There were no significant trends, but a non-significantly elevated RR was shown for the highest category of intensity-weighted exposure days in the fully adjusted model (RR 1.87, 95% CI 0.67 to 5.27). There were no cases of multiple myeloma in glyphosate users with unknown extent of use (see Table footnote for numbers of applicators).

**Table 3:** Risks of multiple myeloma in relation to estimated glyphosate exposure (cumulative exposure days or intensity-weighted exposure days) (32 cases [29 males, 3 females] in 54,315 applicators, Set 1 data **^a^**).

Glyphosate Exposure	*n*	RR ^b^	(95% CI)	RR ^c^	(95% CI)	RR ^d^	(95% CI)
*Cumulative exposure days* **^e^**							
Never used	8	1.0		1.0		1.0	
1–20 days	10	1.06	(0.42 to 2.70)	1.13	(0.44 to 2.88)	1.14	(0.43 to 3.03)
21–56 days	8	1.34	(0.50 to 3.58)	1.50	(0.56 to 4.05)	1.52	(0.54 to 4.34)
57–2678 days	6	1.08	(0.37 to 3.11)	1.23	(0.42 to 3.58)	1.38	(0.42 to 4.45)
*p*-value for trend **^f^**		*p* > 0.50		*p* > 0.50		*p* = 0.48	
*p*-value for trend **^g^**		*p* > 0.50		*p* > 0.50		*p* > 0.50	
*Intensity-weighted exposure days* **^h^**							
Never used	8	1.0		1.0		1.0	
0.1–79.5 units	6	0.91	(0.31 to 2.62)	0.99	(0.34 to 2.86)	1.00	(0.33 to 3.00)
79.6–337.1 units	8	1.12	(0.42 to 3.00)	1.22	(0.45 to 3.28)	1.27	(0.45 to 3.56)
337.2–18,241 units	10	1.44	(0.57 to 3.67)	1.65	(0.64 to 4.24)	1.87	(0.67 to 5.27)
*p*-value for trend **^f^**		*p* = 0.39		*p* = 0.27		*p* = 0.22	
*p*-value for trend **^i^**		*p* = 0.33		*p* = 0.24		*p* = 0.18	

**^a^** Set 1 comprised 54,315 applicators excluding those with any cancers diagnosed before enrolment, applicators lost to follow-up, who had missing data for age at enrolment, or who provided no information on whether they had ever used glyphosate; **^b^** Adjusting for age at enrolment (<50 years, 50–59 years, 60–69 years, ≥70 years), and gender; **^c^** Further adjustment for cigarette smoking, use of alcohol, family history of cancer, and education (for levels see Table 2); **^d^** Further adjustment for use of ten other pesticides (for levels see Table 2); **^e^** Excluding 659 subjects who used glyphosate with unknown exposure estimate; **^f^** Four categories scored 1–4; **^g^** Four categories scored by mean exposures of 0.0, 8.75, 38.75, and 116.0 days respectively; ^h^ Excluding 1011 subjects who used glyphosate with unknown exposure estimate; **^i^** Four categories scored by mean exposures of 0.0, 38.38, 161.7 and 788.8 units (level-days) respectively.

Table 4 shows RR estimates for multiple myeloma in terms of levels of ever-use of glyphosate, reported cumulative days of glyphosate use, and estimated intensity-weighted exposure days for the 55,934 applicators in Set 4 with a total of 34 cases of multiple myeloma. The table summarises the findings of three separate analyses (for the three exposure metrics) with adjustment for age at enrolment, gender, family history of cancer, and level of education. The risk of multiple myeloma in ever-users of glyphosate was close to unity (RR 1.18, 95% CI 0.36 to 8.20) and there were no significant trends with either of the two cumulative exposure metrics. The applicators excluded from the analysis summarised in Table 3 were not excluded from this Table because there were two cases of multiple myeloma in the combined category of “ever-use of glyphosate not known”, and “extent of use not known”.

**Table 4:** Risks of multiple myeloma in relation to estimated glyphosate exposure (ever-use cumulative exposure days or intensity-weighted exposure days) (34 cases (31 males, 3 females) in 55,934 applicators, Set 4 data **^a^**).

Glyphosate Exposure	*n*	RR ^b^	(95% CI)
*Glyphosate use*			
Never	8	1.0	
Ever	24	1.18	(0.53 to 2.65)
not known	2	1.71	(0.36 to 8.20)
*Cumulative exposure days*			
Never used	8	1.0	
1–20 days	10	1.11	(0.44 to 2.83)
21–56 days	8	1.45	(0.54 to 3.88)
57–2678 days	6	1.17	(0.40 to 3.41)
not known **^c^**	2	1.19	(0.25 to 5.65)
*p*-value for trend **^d^**		*p* > 0.50	
*p*-value for trend **^e^**		*p* > 0.50	
*Intensity-weighted exposure days*			
Never used	8	1.0	
0.1–79.5 units	6	0.95	(0.33 to 2.75)
79.6–337.1 units	8	1.19	(0.44 to 3.19)
337.2–18,241 units	10	1.58	(0.62 to 4.05)
not known **^c^**	2	1.04	(0.22 to 4.92)
*p*-value for trend **^d^**		*p* = 0.30	
*p*-value for trend **^f^**		*p* =0.26	

**^a^** Set 4 comprised 54,934 applicators excluding those with any cancers diagnosed before enrolment, applicators lost to follow-up, or who had missing data for age at enrolment; **^b^** Adjusting for age at enrollment (<50 years, 50–59 years, 60–69 years, ≥70 years), gender, family history of cancer, and education (for levels see Table 2); **^c^** Use or extent of use not known; **^d^** First four categories scored 1–4; **^e^** First four categories scored by mean exposures of 0.0, 8.75, 38.75, and 116.0 days, respectively; **^f^** First four categories scored by mean exposures of 0.0, 38.38, 161.7 and 788.8 units (level-days), respectively.

## 4. Discussion

This study found no significant trends of multiple myeloma risk with reported cumulative days of glyphosate use and unexceptional point estimates of risk for ever-use of glyphosate. This was irrespective of whether the analyses had adjustment for a few basic variables (age and gender) or adjustment for many other lifestyle factors or pesticide exposures, as long as data on all available pesticide applicators was used. The suspiciously elevated RRs reported previously [3] arose from the use of restricted data sets that, probably by chance, turned out to be unrepresentative. These restrictions would seem to be unnecessary because there is no technical problem in dealing with missing data in Poisson regression. In addition the restrictions may be undesirable as potentially informative data on the exposure/outcome under investigation are being discarded. To be concrete, Set 2 had 9% fewer applicators than Set 1, but lost 50% (*n* = 4) of multiple myeloma cases in those applicators who had never used glyphosate. Two of these cases were excluded because they were without data on cigarette smoking and the remaining two cases were without data on use of alcohol. Set 3 had 25% fewer applicators than Set 1, but lost 63% (*n* = 5) of multiple myeloma cases in those applicators who had never used glyphosate. These lost cases in the baseline category (never-users) gave a false impression of elevated rates in ever-users. The main reason for carrying out this analysis was to discover whether there are good reasons to give more weight to one of the two point estimates of risk (1.1 and 2.6) for ever-use of glyphosate reported previously. The answer must be the lower estimate, because it is based on the full data set and is, in fact, almost unaffected by simultaneous adjustment for other variables whereas the higher estimate is based on an unrepresentative (biased) dataset. 

The practice of restricting analyses to subjects with complete data for all variables is, perhaps, a procedure to be carried out with caution as it is clear from this example that such restrictions can lead to misleading findings. It also ignores the fact that findings for missing categories can often be interesting in their own right. For example in the AHS the data on smoking have merit because lung cancer risks are related to the reported smoking data. This relationship (predicted to be positive on the basis of other studies [10]) has been calculated by this author as follows: non-smokers RR 1.0 (by definition); ≤12 pack-years RR 3.24, 95% CI 1.47 to 7.15, and >12 pack-years RR 22.1, 95% CI 11.7 to 42.0. What is interesting is that the point estimate of RR is similarly elevated for the “smoking not known” category (RR 26.2, 95% CI 12.7 to 54.1). One possible explanation for this is that the “not known” category is comprised in the main by heavy smokers who are perhaps tired of being asked about their smoking habits. By excluding applicators with unknown smoking history one would not be excluding a random sample of the study cohort but rather a group of heavy smokers. Of course, such exclusion would not in itself produce a biased dataset. Such bias would only occur if the exclusion is related to exposure and outcome as happened here with the loss of an unusual percentage of cases of multiple myeloma in non-users of glyphosate. 

One problem with some of these analyses (Table 2 for example) is that many risk coefficients are being calculated simultaneously from a dataset that only comprises 32 cases. It is perhaps surprising that the fuller analyses have not produced more erratic results. Most attention could be given to the analyses with limited adjustment for other variables (Table 4 for example) because ideally one would like to have only true risk factors or surrogates for true risk factors in the model. 

A 2010 review of all AHS publications concluded that multiple myeloma was not associated with glyphosate exposure in the AHS [16]. Data on multiple myeloma risk and glyphosate use is also available in a later Canadian case-control study of 342 cases of multiple myeloma and 1506 population-based controls [17]. Details of pesticide exposures were collected by telephone interview for those subjects who reported exposure for at least ten hours per year. A non-significantly elevated RR was reported for glyphosate use based on 32 exposed cases (RR 1.22, 95% CI 0.77 to 1.93). A more detailed analysis of these Canadian data found a non-significant deficit in risk for light users (≤2 days per year) of glyphosate (RR 0.72, 95% CI 0.39 to 1.32, 15 exposed cases) and an excess risk that approached statistical significance in heavier users (>2 days per year) (RR 2.04, 95%CI 0.98 to 4.23, 12 exposed cases) [18]. Such results could be obtained if recall bias led some exposed cases to overstate their annual use. Excess risks of multiple myeloma in farmers have been reported in meta-analyses of farm worker studies [12,13]. Possible explanations for such excesses include pesticide exposures and contacts with farm animals. A recent pooled analysis of five case-control studies from North America and Europe into the aetiology of multiple myeloma reported “equivocal results” in supporting or refuting earlier associations with occupational pesticide exposure [19].

This study has the same limitations that have been noted previously [3], namely the relatively small number of cases of multiple myeloma available for analysis, and the absence of information on timing of pesticide exposure (other than before enrolment). In addition this new analysis was not able to adjust for State of residence. Follow-up data continues to accrue in the AHS and a total of 71 cases of multiple myeloma have now been identified to the end of 2006 in private applicators [20]. It will be important to use these data together with extended follow-ups to continue to monitor cancer risks in glyphosate users and other pesticide applicators and it is hoped that the current paper will encourage the use of full datasets rather than restricted datasets.

## 5. Conclusions

This secondary analysis of AHS data does not support the hypothesis that glyphosate use is a risk factor for multiple myeloma, and suggests that the practice of restricting analyses to subjects with complete data for all variables is perhaps not to be recommended.
